# The Effectiveness of Financial Incentives for Health Behaviour Change: Systematic Review and Meta-Analysis

**DOI:** 10.1371/journal.pone.0090347

**Published:** 2014-03-11

**Authors:** Emma L. Giles, Shannon Robalino, Elaine McColl, Falko F. Sniehotta, Jean Adams

**Affiliations:** 1 Institute of Health and Society, Newcastle University, Newcastle upon Tyne, Tyne and Wear, United Kingdom; 2 Newcastle Clinical Trials Unit, Institute of Health and Society, Newcastle University, The Medical School, Framlington Place, Newcastle upon Tyne, Tyne and Wear, United Kingdom; Iran University of Medical Sciences, Iran (Islamic Republic of)

## Abstract

**Background:**

Financial incentive interventions have been suggested as one method of promoting healthy behaviour change.

**Objectives:**

To conduct a systematic review of the effectiveness of financial incentive interventions for encouraging healthy behaviour change; to explore whether effects vary according to the type of behaviour incentivised, post-intervention follow-up time, or incentive value.

**Data Sources:**

Searches were of relevant electronic databases, research registers, www.google.com, and the reference lists of previous reviews; and requests for information sent to relevant mailing lists.

**Eligibility Criteria:**

Controlled evaluations of the effectiveness of financial incentive interventions, compared to no intervention or usual care, to encourage healthy behaviour change, in non-clinical adult populations, living in high-income countries, were included.

**Study Appraisal and Synthesis:**

The Cochrane Risk of Bias tool was used to assess all included studies. Meta-analysis was used to explore the effect of financial incentive interventions within groups of similar behaviours and overall. Meta-regression was used to determine if effect varied according to post-intervention follow up time, or incentive value.

**Results:**

Seventeen papers reporting on 16 studies on smoking cessation (n = 10), attendance for vaccination or screening (n = 5), and physical activity (n = 1) were included. In meta-analyses, the average effect of incentive interventions was greater than control for short-term (≤six months) smoking cessation (relative risk (95% confidence intervals): 2.48 (1.77 to 3.46); long-term (>six months) smoking cessation (1.50 (1.05 to 2.14)); attendance for vaccination or screening (1.92 (1.46 to 2.53)); and for all behaviours combined (1.62 (1.38 to 1.91)). There was not convincing evidence that effects were different between different groups of behaviours. Meta-regression found some, limited, evidence that effect sizes decreased as post-intervention follow-up period and incentive value increased. However, the latter effect may be confounded by the former.

**Conclusions:**

The available evidence suggests that financial incentive interventions are more effective than usual care or no intervention for encouraging healthy behaviour change.

**Trial Registration:**

PROSPERO CRD42012002393

## Introduction

Despite consistent efforts to encourage uptake of healthy behaviours [1], [2], unhealthy behaviours remain common in developed countries [3]. Financial incentives have been suggested as one method of promoting healthy behaviour change.

Individual decisions to engage in behavioural options are influenced by beliefs about the likely consequences of performing those behaviours [4]. Individuals commonly hold inconsistent preferences for outcomes occurring at different points in the future, and for outcomes that are more or less certain. In general, outcomes that will occur in the near future or with more certainty, are valued more than those in the distant future or with less certainty [4]. Whilst anticipated health gains of healthy behaviours are often delayed in time and are uncertain (e.g. reduced risk of disease in the future), the financial and opportunity costs can be immediate and certain (e.g. giving up leisure time to take part in physical activity) [5]. As these immediate, certain costs are often ‘dis-valued’ more than the delayed, uncertain health benefits are valued, individuals make a ‘rational’ choice to pursue unhealthy behaviours. It is hypothesised that health promoting financial incentive interventions (HPFI) provide near-immediate and certain rewards for, or reduce the immediate costs of, health-promoting behaviours, and so change the reward structure associated with these behaviours making them more attractive to individuals [5].

The complexities of HPFI and the challenges of defining them have been previously acknowledged [6], [7]. However, incentive interventions share in common that they offer motivating rewards contingent on behavioural performance [6], [8]. Here we define HPFI as cash or cash-like rewards (e.g. vouchers that can be exchanged for goods or services) or penalties (e.g. reductions in welfare benefits), provided contingent on performance of healthy behaviours.

It is commonly suggested that HPFI are more useful for encouraging simple one-off behaviours, such as attendance for vaccinations, than more complex sustained behaviour change, such as smoking cessation [9]–[11]. However, we are not aware of any systematic evidence synthesis that has arrived at this conclusion, but it may be related to a common concern that the effects of HPFI diminish quickly after incentives are withdrawn [12], [13], meaning that any behaviour change achieved is unlikely to be sustained. A previous review did conclude that external rewards can reduce an individual's internal motivation to pursue behaviour change, such that they become dependent on the reward, rather than any personal desire to pursue the healthy behaviour [14]. However, this finding is based on laboratory-based research and may not be generalisable to community settings.

Some authors have suggested that HPFI may be more suitable for, or attractive to, individuals living in more deprived circumstances [15], [16]. However, variations in effectiveness of HPFI across population groups have not been systematically explored. Overall, little is known about what makes an effective HPFI, in terms of value, format or other characteristics of the incentive, behaviour, or recipient.

A number of reviews, using both systematic and other methods, have now been conducted on the effects of HPFI [11], [17]–[23]. However, these focus on single, specific behaviours rather than exploring the full range of healthy behaviours [17], [21]–[23]; are restricted to developing countries where absolute financial hardship may be more common than in developed countries [20]; or use non-systematic methods for searching and screening, meaning that findings may be biased [11], [18], [19].

We aimed to fill the gaps identified by conducting a systematic review of primary studies exploring the effectiveness of HPFI compared to non-intervention or usual care, to encourage uptake of any healthy behaviours, in non-clinical adult populations living in high-income countries. We also explored whether the effects of HPFI varied according to the type of behaviour incentivised, follow-up time after incentive withdrawal, or the value or format of the incentive itself.

## Methods

The protocol (see File S1) for this review was published in full [24] and registered with PROSPERO before searching commenced (Registration no. 2012:CRD42012002393). Although a number of the original research questions could not be answered due to limited data availability, there were no substantive variations from the protocol. The review is reported according to the Preferred Reporting Items for Systematic Reviews (PRISMA) guidelines (see Checklist S1) [25].

### Information sources

Relevant electronic databases of peer-reviewed literature were searched from the earliest date available to April 2012. These were: Medline, Embase, Science Citation Index, Cumulated Index to Nursing and Allied Health Literature (CINAHL), Social Science Citation Index, PsycINFO, Applied Social Science Index and Abstracts, International Bibliography for the Social Sciences and The Cochrane Library (including DARE, CENTRAL, HTA, and NHS EDD). The search strategy combined relevant terms for ‘incentives’, ‘behaviour’ and ‘behaviour change’. An example of the full electronic search strategy used in MEDLINE is provided in File S2 and this was adapted, as appropriate, for other databases.

Manual searches of online research registers (Current Controlled Trials, clinicaltrials.gov) were conducted alongside searches of www.google.com. Relevant National Academic Mailing List groups (Jiscmail) were also sent requests for relevant information. The reference lists of relevant previous reviews [11], [17], [18], [21]–[23], [26]–[46] and all papers meeting the inclusion criteria [47]–[63] were also reviewed. Citation searches of included papers were conducted using Science Citation Index and Social Science Citation Index.

Endnote ×6 was used to manage search results.

### Eligibility criteria

We searched for published and unpublished controlled evaluations of the effectiveness of HPFI, compared to no intervention or usual care, to encourage uptake of healthy behaviours in non-clinical adult populations, living in high-income countries. The inclusion criteria are described in full in Table S1. The inclusion of all controlled study designs was as suggested by the Cochrane Effective Practice and Organisation of Care Group (http://epoc.cochrane.org/epoc-resource). In order to focus on the effect of financial incentives on health behaviour change, only studies that had behavioural outcomes were included. Studies that used process markers of change only (e.g. weight loss, but not physical activity or diet) were excluded. We restricted the review to studies measuring behaviour change using objective, or validated self-reported, methods to ensure high levels of validity. By ‘validated self-report’ we mean non-objective measures that have previously been reported to be valid compared to an objective measure. We restricted the review to non-clinical populations to ensure applicability to behaviour change in free-living ‘healthy’ adults and so ensure maximum public health applicability. Many ‘incentive’ schemes offer participants a non-guaranteed reward for behaviour change – e.g. entry into a lottery. Individuals differ in their conceptualisations of risk and uncertainty, and so we restricted the review to HPFI interventions provided with 100% certainty to ensure that such differences did not confound the results.

### Study selection and data collection

After exclusion of duplicates, the title and abstract of all retrieved papers were screened by one researcher (ELG) to exclude obviously irrelevant papers. The full texts of remaining papers were independently screened by two researchers (ELG & JA) to identify those meeting the inclusion criteria. Any disagreements were resolved by discussions.

Data was extracted independently by two researchers (ELG & JA) using a pro-forma developed for this purpose. Extracted information included: bibliographic details, information on participants, HPFI interventions, comparators, outcomes, study design, and results. Incentive interventions were described using a framework for this purpose [6]. Disagreements were resolved by discussion.

The inclusion criteria restricted HPFI to those that would definitely be provided if behaviour change occurred. The value of these certain HPFI, over and above participant payments, were identified and converted into 2011 US$ to allow comparisons between studies (http://www.measuringworth.com/ppowerus/). Uncertain, chance incentives (e.g. entry into lotteries), were offered alongside certain incentives in some studies. As the probability of winning, and the value of winnings, was often not clear, these were not included in calculations of total incentive value.

### Risk of bias

Risk of bias in included studies was assessed independently by two researchers (ELG & JA) using the Cochrane Risk of Bias Review Guidelines [64]. Disagreements were resolved by discussion.

### Synthesis of results

Studies examining the effects of HPFI for similar behaviours were grouped together in a tabular summary for narrative synthesis.

Incentives were described, using a framework for this purpose [6], in terms of: direction (reward or penalty), form (cash, vouchers or goods), magnitude, whether incentives were certain only or also included chance components, target behaviour, frequency of reward (all or some instances incentivised), immediacy of reward in relation to behaviour, schedule (fixed or variable), and recipient(s) of incentives (individuals or groups). Where studies reported more than one relevant comparison (e. g. multi-arm trials comparing a number of different incentive values to control), all were identified and described.

For all groups of behaviours where more than one relevant comparison was present and sufficient data were available, meta-analysis was undertaken by group. Where more than one intervention arm from a single study was included in a single meta-analysis, the control group was divided in proportion to the relative sizes in each intervention arm to avoid double counting [65]. Many studies on smoking cessation included a number of different follow-up points. Meta-analyses of smoking cessation studies were performed for medium (≤six months) and longer term (>six months) follow-up points and included only the longest follow-up point in each category from each study.

In addition to meta-analyses by behavioural group, an overall meta-analysis including all comparisons from all included studies, where data was available, was also performed. This was restricted to only the longest follow-up point from studies including multiple follow-ups.

Throughout, random-effects meta-analysis was conducted using Cochrane Collaboration Review Manager 5.1. Risk ratios (RR) and 95% confidence intervals (CI) were calculated for use in forest plots. Where there was evidence of a high level of heterogeneity (i.e. I^2^>75%) [66], further sub-group analyses by intervention design were explored. Contour enhanced funnel plots were drawn using Comprehensive Meta-Analysis 2.0 to assess potential publication bias.

Meta-regression was conducted within the same groups as meta-analyses, to explore whether log transformed study RR varied by incentive value, or (where appropriate) follow-up period. No other characteristics of interventions or participants were reported consistently enough to allow exploration of the effects of these on RR. Unrestricted maximum likelihood mixed-effects meta-regression was conducted in Comprehensive Meta-Analysis 2.0 and meta-regression plots, with points proportional in size to comparison weights drawn.

## Results

**Figure 1:**
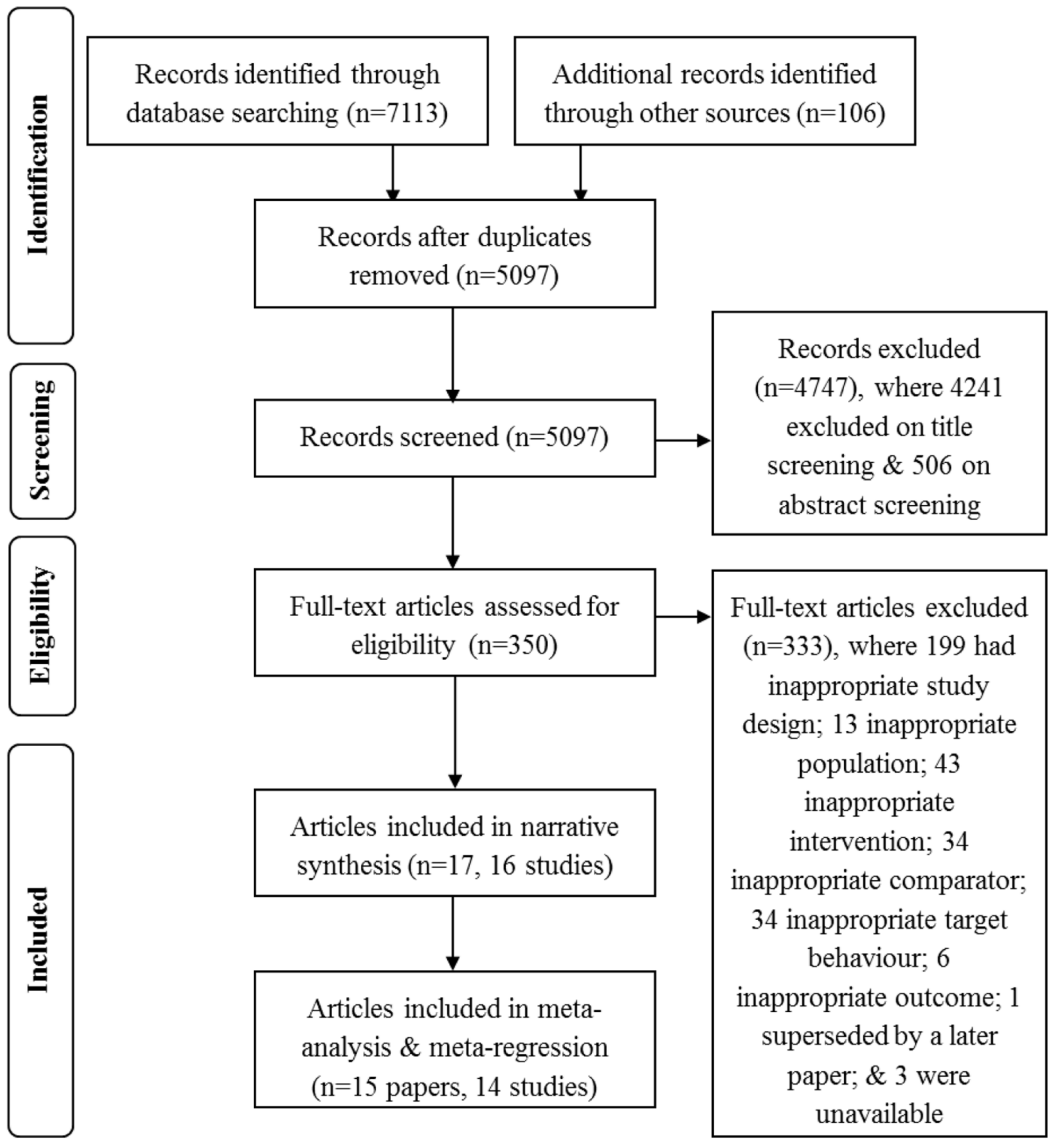
Flow diagram of study selection and exclusion.

The full text of three papers that were potentially relevant could not be located and were excluded from the review [67]–[69]. The full text of 350 papers were screened and a total of 17 papers met the inclusion criteria and were included in the review (Figure 1). Two papers reported data from different follow up points for the same study, leaving 16 included studies [51], [52].

The characteristics of included studies are summarised in Table S2. Of the 16 studies, ten studies focused on smoking cessation [48], [50]–[54], [59]–[63], five on attendance for vaccination or screening [47], [55]–[58], and one on physical activity [49]. All included studies were randomised controlled trails (RCTs) or cluster RCTs. All 14 studies which provided information on location were conducted in the USA [47]–[56], [58], [61]–[63]. Authors of the remaining studies were based in the USA and it is likely that participants were too [57], [59], [60].

Most HPFI offered were cash rewards [48], [49], [51], [52], [57], [59]–[63] and/or vouchers exchangeable for a specific range of goods or services [47], [55], [56]. Two studies [53], [54] used deposit contracts where participants made cash deposits at the start of the study which were only returned in the event of successful behaviour change – resulting in potential financial penalties. Two studies also included additional uncertain rewards contingent on behaviour change (e.g. entry into lotteries) in addition to certain rewards [50], [58].

The total value of certain financial incentives that study participants could receive for successful behaviour change, over and above any payments for study participation, ranged from $5.16 [57], to $786 (in 2011 $US) [62].

Intervention periods in the smoking cessation studies ranged from two weeks [59], [60], to 24 months [54], with post-intervention follow-up periods ranging from four weeks [59], [60] to 24 months [50]–[52], [54]. Most studies on attendance for vaccination or screening involved a reward for one-off attendance with no prolonged intervention or follow-up period [47], [55]–[57]. One study assessed repeated attendances for a series of injections over a 24 week period with incentives provided for each attendance [58]. The physical activity study had an intervention period of four weeks with final follow-up immediately following the intervention period [49].

**Figure 2:**
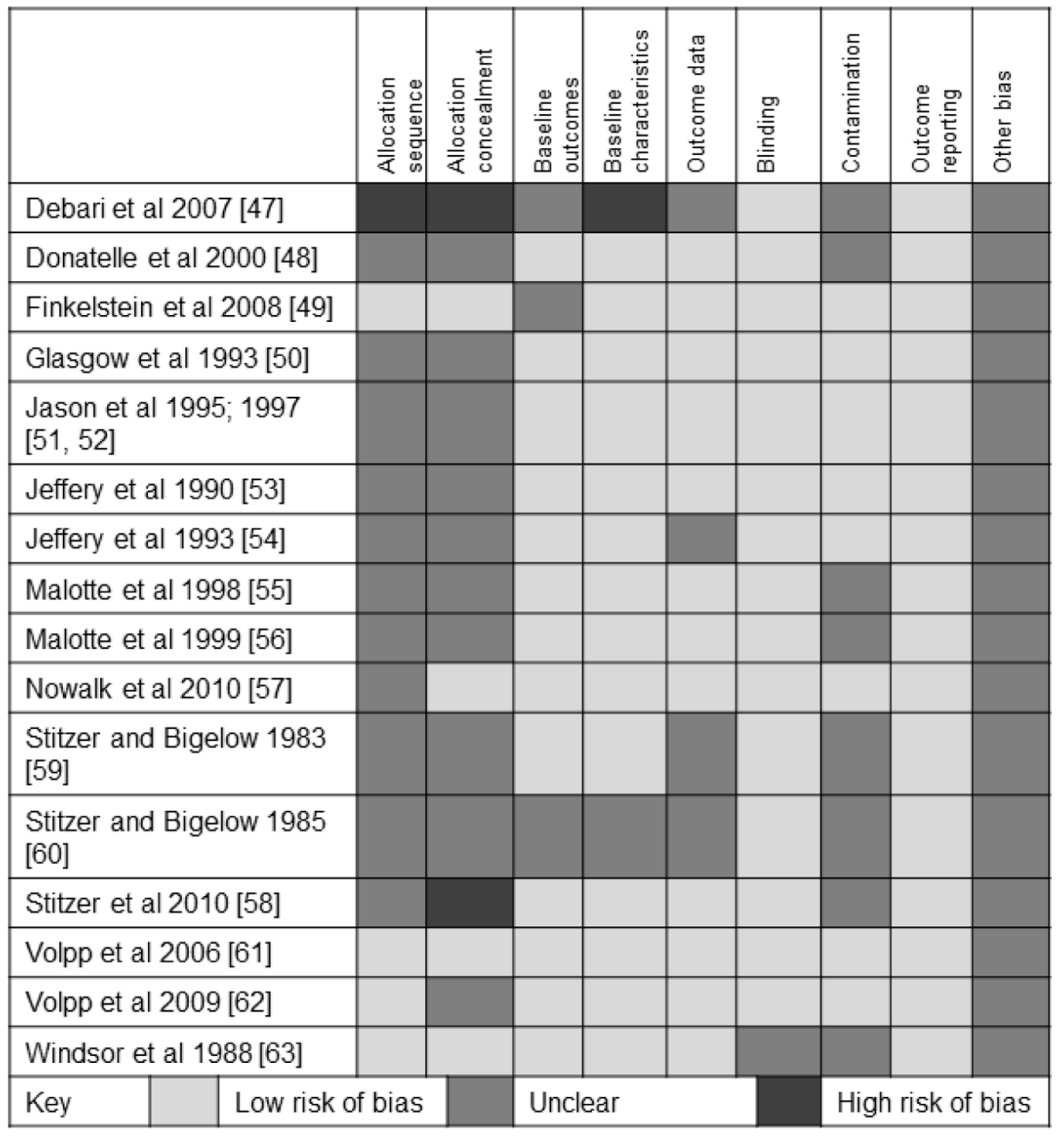
Risk of bias in included studies.

The risk of bias in included studies was low or unclear in most areas in most studies (Figure 2). Allocation sequence and allocation concealment, together with possible selection bias (the main source of ‘other’ bias arising from using volunteer samples), were the main potential sources of bias. The risk of bias was high in relation to allocation sequence, allocation concealment, and baseline characteristics in one study [47]. This study was not included in meta-analyses or meta-regression due to insufficient data being presented on numbers of participants in each group, and details of outcomes in each group. Attempts to contact the authors were unsuccessful.

Overall, 15 of the 16 studies met the criteria for inclusion in meta-analyses and meta-regressions: all ten of the studies on smoking cessation, four out of five of the studies on attendance for vaccination and screening, and the single study on physical activity – although this latter study was only included in the analysis of all behaviours combined. The remaining study on attendance for screening was excluded due to insufficient data [47].

### Smoking cessation

**Figure 3:**
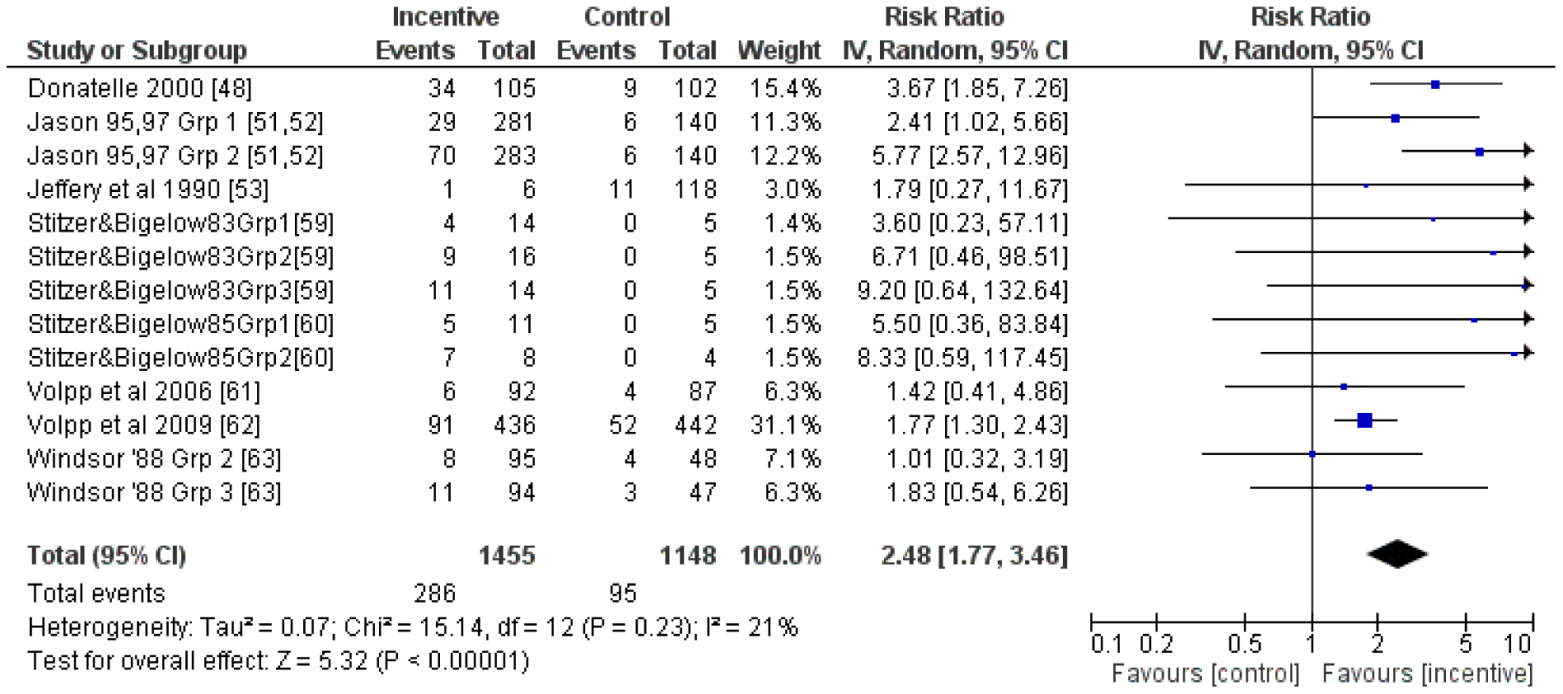
Meta-analysis of financial incentives for smoking cessation (follow-up ≤six months).

**Figure 4:**
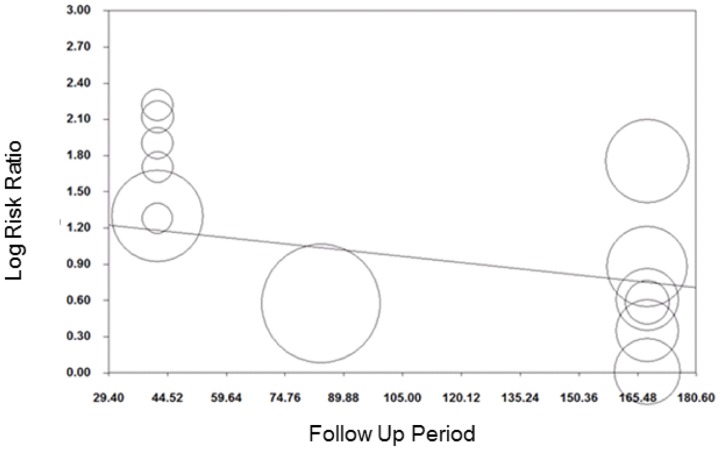
Meta-regression of follow-up period on relative risk, smoking cessation (follow-up <six months).

**Figure 5:**
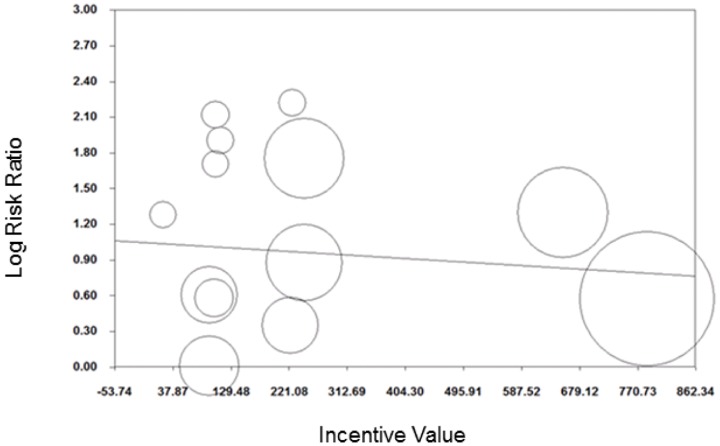
Meta-regression of incentive value on relative risk, smoking cessation (follow-up up <six months).

Meta-analyses of 13 comparisons from eight studies on smoking cessation which reported outcomes ≤six months follow-up, revealed an average RR (95% CI) of 2.48 (1.77 to 3.46) (Figure 3) in favour of incentives. An *I^2^* of 21%, indicating low evidence of heterogeneity [66], was not explored further. Meta-regression of this group of studies revealed no evidence that study RR was associated with follow-up time (beta (95%CI): −0.003 (−0.01 to 0.003); Figure 4) or total incentive value (beta (95%CI): −0.003 (−0.001 to 0.0008); Figure 5).

**Figure 6:**
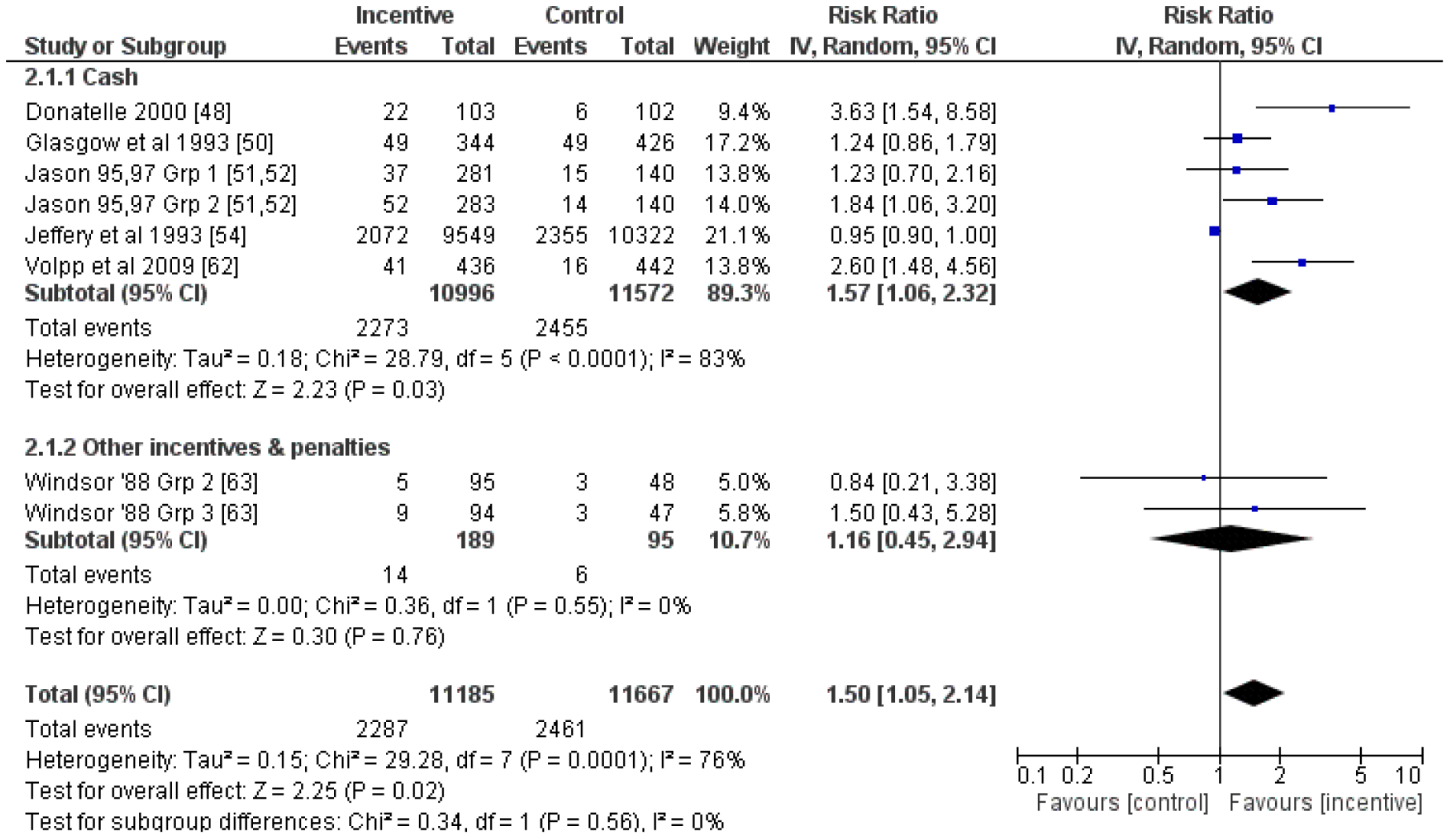
Meta-analysis of financial incentives for smoking cessation (follow-up >six months).

Six studies, including eight comparisons, were included in meta-analysis of the effect of financial incentives for smoking cessation for follow-ups >six months. This revealed an average RR (95% CI) of 1.50 (1.05 to 2.14) (Figure 6). An *I^2^* of 76% indicated high evidence of heterogeneity. Subgroup analyses suggested that the average effect of cash-only financial incentives (RR (95%CI): 1.57 (1.06 to 2.32) was greater than that for other formats (RR (95%CI): 1.16 (0.45 to 2.94) and that the latter was not statistically significant. Although high heterogeneity remained in the cash-only sub-group (*I^2^* = 83%), all other sub-group analyses resulted in inclusion of groups containing only one comparison.

**Figure 7:**
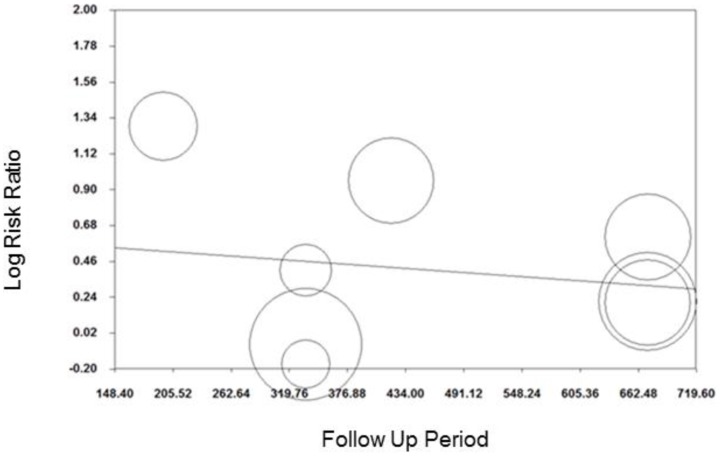
Meta-regression of follow-up period on relative risk, smoking cessation (follow-up >six months).

**Figure 8:**
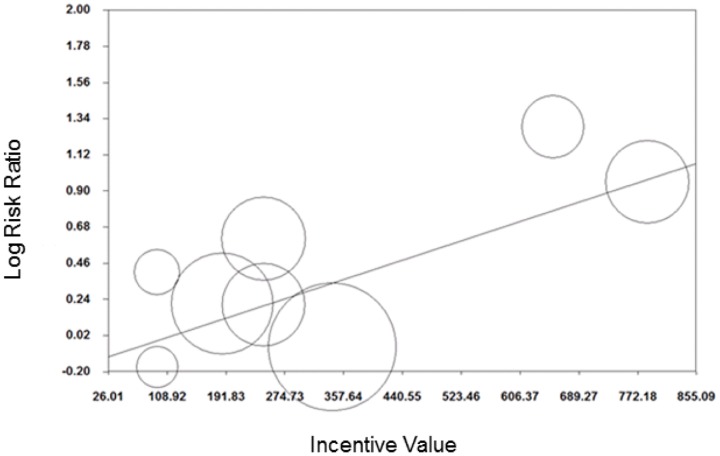
Meta-regression of incentive value on relative risk, smoking cessation (follow-up of >six months).

Meta-regression showed no evidence that the log transformed RR of financial incentives for smoking cessation with >six months follow-up varied by follow-up period (coefficient (95%CI): 0.0005 (−0.002 to 0.001); Figure 7). However there was some evidence that log transformed RR increased as incentive value increased (coefficient (95%CI): 0.001 (0.0002 to 0.003); Figure 8).

**Figure 9:**
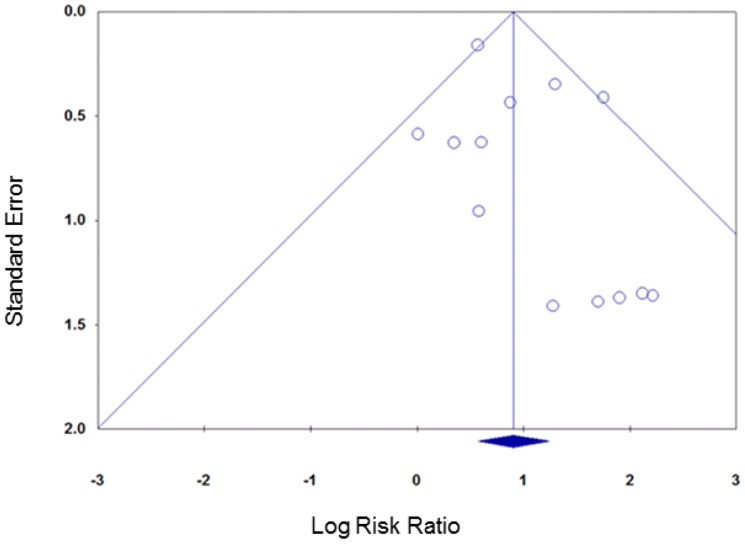
Contour enhanced funnel plot, smoking cessation (follow-up ≤six months).

**Figure 10:**
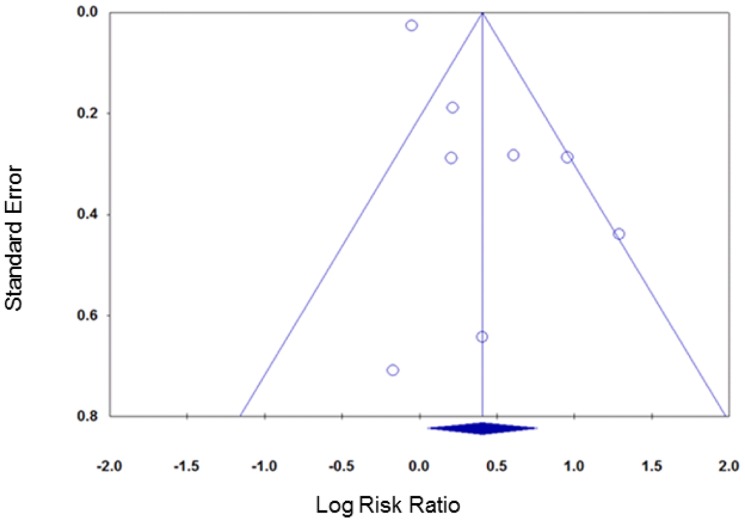
Contour enhanced funnel plot, smoking cessation (follow-up >six months).

Contour enhanced funnel plots did not suggest any funnel plot asymmetry for either group of smoking cessation comparisons (Figure 9 and Figure 10) meaning that the risk of publication bias was low.

### Attendance for vaccination or screening

Of the five studies reporting on the use of financial incentives for increasing attendance for vaccination and screening, one focused on attendance at breast and cervical screening [47], two on attendance for tuberculosis (TB) skin test reading [55], [56] and one each on attendance for influenza and hepatitis B vaccination [57], [58].

**Figure 11:**
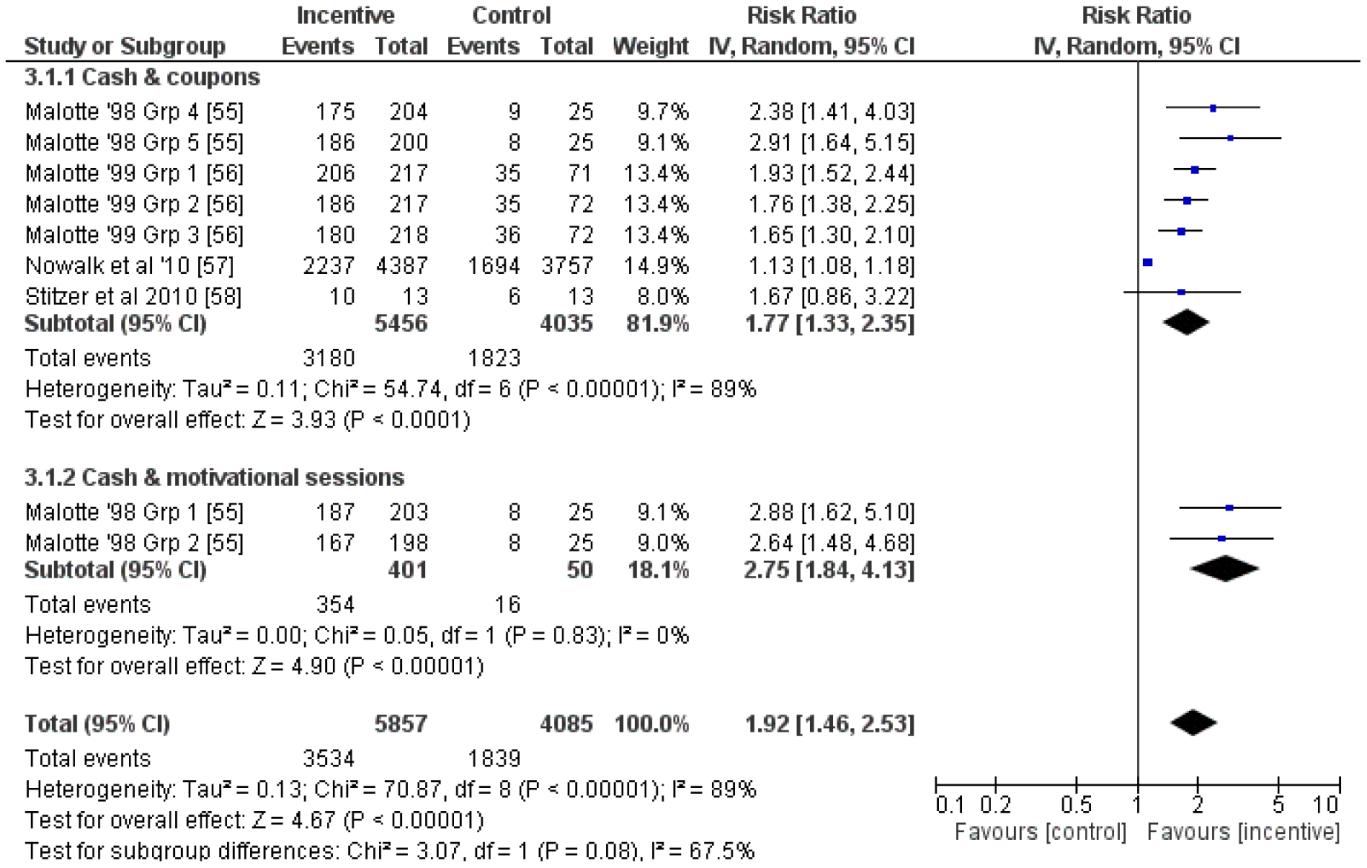
Meta-analysis of financial incentives for attendance at vaccination and screening.

Nine relevant comparisons from four studies were included in a meta-analysis. The average RR (95%CI) was 1.92 (1.46 to 2.53) (Figure 11) with evidence of a high level of heterogeneity (*I^2^* = 89%). Sub-group analyses suggested that cash plus other motivational components (RR (95%CI): 2.75 (1.84 to 4.13)) may be more effective than cash or vouchers alone (RR (95%CI): 1.77 (1.33 to 2.35)). Considerable heterogeneity remained in one subgroup (*I^2^* = 89%) but other approaches to subgroup analyses resulted in subgroups containing only one comparison and were not pursued.

**Figure 12:**
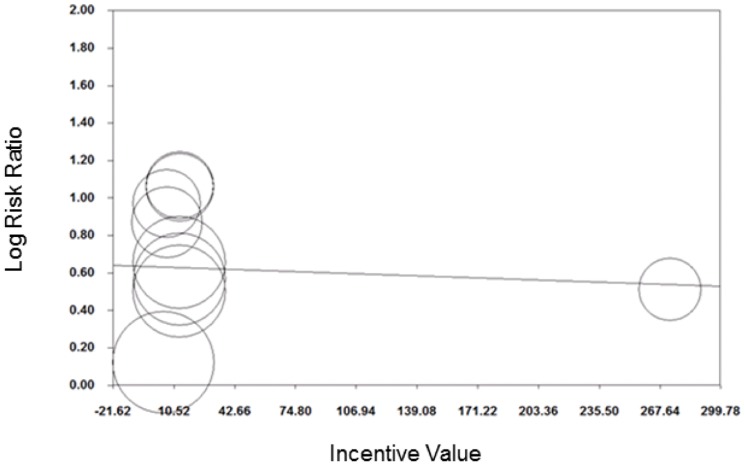
Meta-regression of incentive value on relative risk, attendance at vaccination & screening.

**Figure 13:**
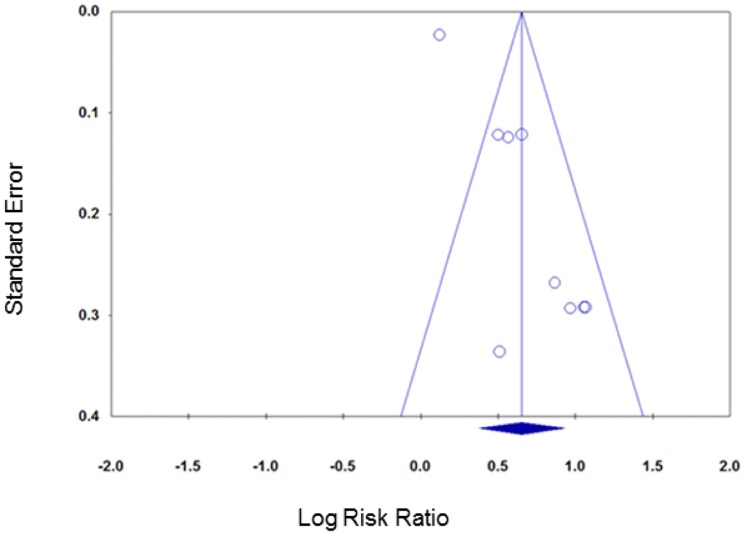
Contour enhanced funnel plot, attendance at vaccination and screening.

Meta-regression revealed no evidence that log transformed RR varied by incentive value (coefficient (95%CI): −0.0004 (−0.004 to 0.003); Figure 12). However, visual inspection of Figure 12 shows minimal variation in incentive values offered. A contour enhanced funnel plot did not suggest any evidence of publication bias (Figure 13).

### Physical activity

Only one relevant comparison was included on physical activity and meta-analysis was not undertaken for this behavioural group [49]. This study used pedometers to measure average daily physical activity over one-week periods and rewarded increases in physical activity with increasing cash incentives. Over the four week intervention period, participants in the financial incentive arm took part in an average of 16 more minutes of physical activity per day than those in the control arm. This difference was statistically significant.

### All behaviours

**Figure 14:**
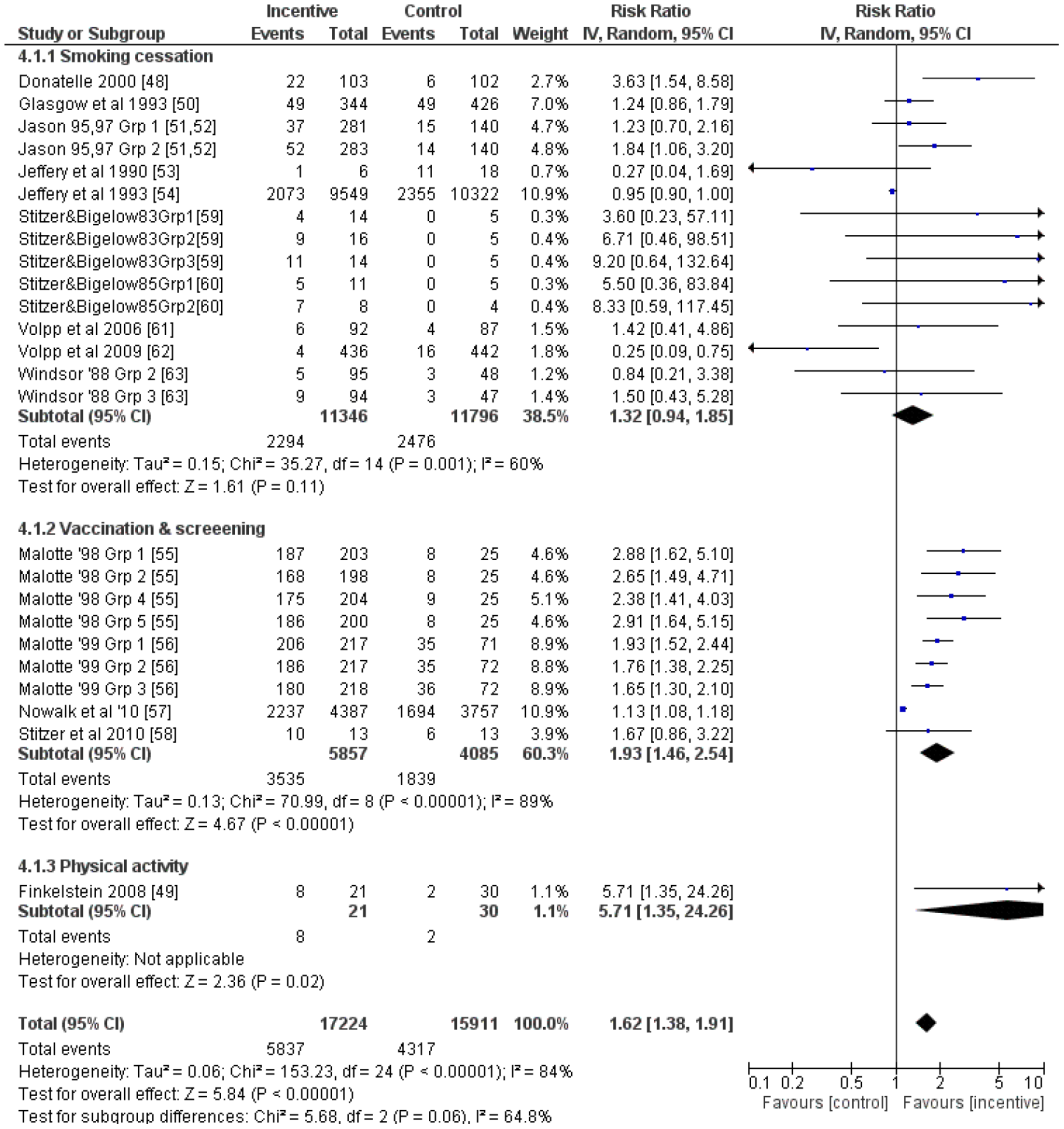
Meta-analysis of financial incentives for all behaviours (latest follow-up point).

A total of 25 relevant comparisons were included in a meta-analysis of all behaviours that included only the longest follow up point from studies with multiple follow-ups. The average RR (95%CI) was 1.62 (1.38 to 1.91) (Figure 14). Although an *I^2^* of 84% suggested considerable heterogeneity, this was not explored further.

**Figure 15:**
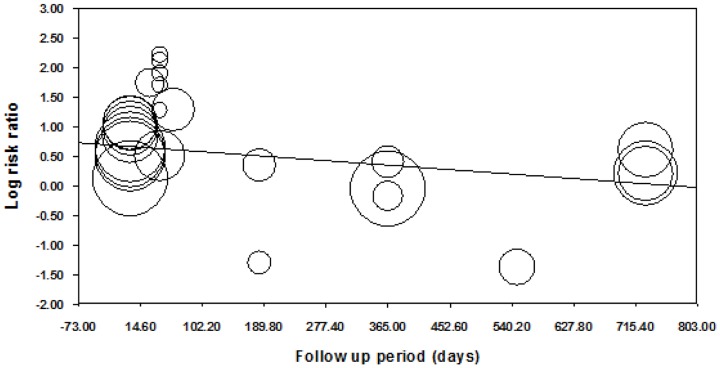
Meta-regression of follow-up point on relative risk, all behaviours (latest follow-up point).

**Figure 16:**
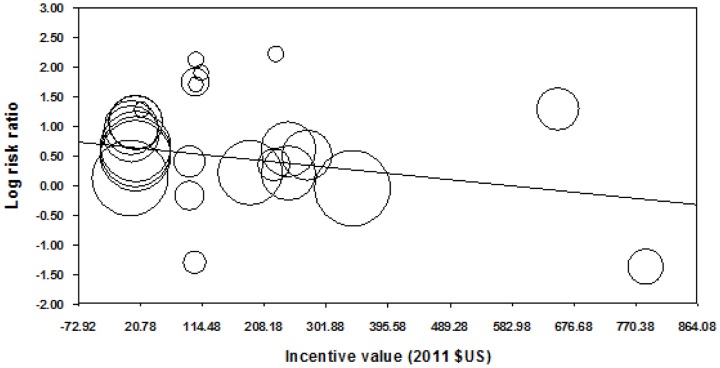
Meta-regression of incentive value on relative risk, all behaviours (latest follow-up point).

**Figure 17:**
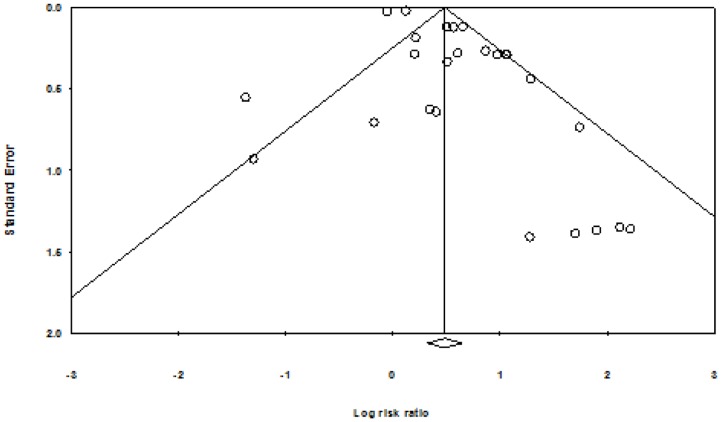
Contour enhanced funnel plot, all behaviours (latest follow-up point).

Meta-regression showed some evidence that log transformed RR decreased as post-intervention follow-up period increased (coefficient (95%CI): −0.001 (−0.002 to −0.0002); Figure 15) and incentive value increased (coefficient (95%CI): −0.001 (−0.002 to −0.0001); Figure 16). The funnel plot did not suggest clear evidence of publication bias (Figure 17).

## Discussion

### Statement of principal findings

This is the first systematic review which brings together evidence on the effectiveness of financial incentive interventions for encouraging uptake of the full range of health promoting behaviours in non-clinical adult populations living in high-income countries. A total of 17 papers reporting on 16 studies met the inclusion criteria and were included in the review. These explored the effect of HPFI on smoking cessation, attendance for vaccinations or screening, and physical activity. Overall, this review found evidence that HPFI were more effective than no intervention, or usual care, in changing behaviours. This was seen for groups of similar behaviours (i.e. smoking cessation, attendance for vaccinations or screening) as well as when all behaviours were combined. There was no clear evidence that HPFI were more effective for ‘simple’ behaviours (e.g. attendance for vaccination or screening) than ‘complex’ ones (e.g. smoking cessation). Financial incentive interventions took a range of formats and it was difficult to draw conclusions on the most effective of these, particularly given the lack of detailed information on the exact nature of interventions and study participants, as well as the absence of trials which have sought to determine if effects of interventions vary according to socio-demographic characteristics. When all behaviours were grouped together, there was some evidence that effect decreased as post-intervention period increased and as total incentive value increased. However, it is possible that the latter effect was confounded by the former.

### Strength and weaknesses of studies in this review

This review found few controlled studies exploring the effect of HPFI. The studies that were found were restricted to a small number of behaviours. Further, primary, controlled studies exploring the effect of financial incentives on change in a range of other health-related behaviours are required.

The studies included did not appear to be at high risk of bias, but there were some areas that were consistently at greater risk of bias, particularly: allocation sequence generation and allocation concealment. In most cases, information was not provided, rather than it being clear that methods were weak. Future researchers should consistently report trials according to existing reporting guidance [70].

It appears that all 16 studies included in the review were US-based, potentially limiting generalisability to other cultures and contexts. Further studies based in other countries are required to confirm that the effects reported here are generalisable to other contexts.

The meta-regression plots revealed some gaps in the range of incentive values and follow-up periods that have been explored. In particular, few studies explored medium-size incentives (e.g. $40–250) for encouraging attendance for vaccination and screening; and few studies had post-intervention follow up periods beyond six months.

Although we originally intended to explore if the effect of HPFI varied according to recipient characteristics (e.g. age, gender, socio-economic position), data was not reported in such a way to allow this. Many other public health interventions are differentially effective according to socio-demographic characteristics of participants [71]. Further research is required to determine if HPFI are particularly effective in some population groups.

### Strengths and weaknesses of this review

This is the first systematic review and meta-analysis, that we are aware of, that has explored the effect of financial incentives across the full range of healthy behaviours in non-clinical settings in high-income countries. Previous reviews have either focused on single health behaviours [17], [21]–[23], failed to use standard systematic review methods [11], [18], [34], or have been limited to low and middle-income countries [20].

Restriction to controlled study designs is recommended to minimise risk of bias in conclusions and so increase confidence in results (http://epoc.cochrane.org/epoc-resources). However, it has been argued that other study designs can also contribute useful information to reviews and an alternative approach to evidence synthesis, for example a realist synthesis exploring the context, mechanisms and outcomes of effective components of financial incentive interventions for health behaviour change [72], may help shed additional light on the financial incentive field. The strict inclusion criteria, such as only including studies with objective, or validated self-report behavioural outcome measures and our focus on behaviour change (e.g. physical activity) rather than proxies of this (e.g. body weight), further adds to the confidence in the results; but similarly limits the number of studies meeting the inclusion criteria.

Only studies comparing HPFI to usual care or no intervention were included. Thus, the results indicate the effect of financial incentives compared to minimal intervention. It is not, therefore, clear how HPFI compare to other interventions. Given the controversy associated with HPFI [73]–[76], society may prefer to avoid the widespread use of HPFI if similarly effective alternative interventions are available.

We used an extensive search strategy, including database searches, expert recommendations of studies, and reference and citation searches. As such, we are confident that we are unlikely to have missed any relevant studies. However, it is difficult to conclusively confirm this. In particular, three studies were not fully screened for inclusion as the full papers could not be retrieved [67]–[69].

We found considerable heterogeneity within some meta-analyses. This likely reflects differences in methods, populations, and interventions and is a reality of the type of intervention we were studying [77]. We clearly presented the heterogeneity found, whilst trying to choose appropriate sub-groups to limit it. We also ensured that inclusion criteria were robust and checked that the data was correct where in doubt (by contacting authors) before undertaking meta-analyses [78].

### Interpretation of findings and comparison to previous findings

Unlike previous non-systematic reviews [11], [18], [34], we used well-recognised systematic review methodology with clear inclusion criteria, substantially reducing the risk of bias in our findings. Unlike previous systematic reviews [17], [21]–[23], our inclusion criteria covered the full range of healthy behaviours in non-clinical adult populations living in high-income countries. Together, these represent significant improvements on previous reviews.

Similar to previous findings, we found evidence that financial incentives are effective at encouraging health-promoting behaviours [21], [23], [34]. In the meta-regression including all behaviours, there was some evidence that effectiveness may decrease over time post intervention period. This has previously been reported [17], [21], [23]. However, statistically significant effects persisted at least until six months post-intervention follow-up in smoking cessation studies, suggesting that effects do not suddenly drop off once incentives are withdrawn. Many health promotion interventions are associated with behaviour change in the short, but not longer, term [79] and this problem is not unique to HPFI.

Previous authors have suggested that HPFI may be more effective in changing one-off health behaviours (such as attendance for vaccination and screening) than more complex behaviours (such as smoking) [9], [11], [12], [23]. This study did not find convincing evidence of this with the average RR of incentives for attendance at vaccinations and screening (RR 1.92 (1.46 to 2.53) being less than that for medium term smoking cessation (RR 2.48 (1.77 to 3.46), but greater than that for longer term smoking cessation (RR 1.50 (1.05 to 2.14). It is possible that the distinction between ‘one-off’ and ‘complex’ behaviours is a false dichotomy in the context of HPFI – with incentives for smoking cessation rewarding a series of one-off behaviours. However, McEachan et al (2010) provide evidence that smoking and attending for screening are considered conceptually different on a number of dimensions by both ‘experts’ and members of the public [80]. Further work could usefully explore whether the effectiveness of HPFI varies according to the behavioural dimensions identified by McEachan et al (2010) – but not enough data was available for such analysis in the current review.

Meta-regression of all behaviours combined showed some evidence that effects decreased as incentive value increased. As incentive value was positively correlated with longest post-intervention follow-up point (r = 0.44, p = 0.03), the finding in relation to incentive value may be confounded by that related to follow-up period. Unfortunately we were not able to conduct multi-variate meta-regression to take account of this. Furthermore, we found a positive relationship between inventive value and effect size in smoking cessation studies with follow up of >six months, suggesting that this effect is not consistent. Very weak evidence in favour of larger value HPFI being more effective has been previously reported [21]. However, other authors have suggested that larger incentive values may be interpreted by recipients as reflecting that the behaviour incentivised is somehow ‘risky’ and thus that a payment is needed to offset this [81]. There are likely to be complex relationships between incentive value and characteristics of both incentivised behaviours and recipients that require more detailed exploration.

Similar to previous reviews, we also found that the majority of studies were US-based [34], and that insufficient evidence is currently available to determine optimal incentive value, or format for changing health behaviours [18]. The average RR reported here are larger than some previously reported. For example, in their original review of incentives as well as competitions for smoking cessation, Cahill & Perera (2008) reported a pooled odds ratio (95%CI) at six months follow up of 1.44 (1.01 to 2.04) [17], (pooled odds ratios are not reported in the updated version of this review) [82]. The comparable figures for the results in this review were 3.12 (1.95 to 4.97) (note this is an odds ratio for comparability, but the figures shown elsewhere are risk ratios). However, this previous review included both competitions, as well as incentives, and these differences in inclusion criteria may explain the differences in results found.

### Implications for policy and practice

Financial incentives may be a useful addition to the behavioural change toolkit, particularly for encouraging smoking cessation and attendance for vaccination and screening. We did not find convincing evidence that HPFI work better for changing short-term, one-off behaviours than longer term, more complex behaviours and HPFI should be considered across the spectrum of healthy behaviours. Although there has been some previous concern that the effects of HPFI may be short-lived once incentives are withdrawn, we did not find convincing evidence of this. Nor did we find convincing evidence that larger incentive values are associated with greater behaviour change. This suggests that small incentives may be effective, although it is not clear from our results if larger incentives produce larger effects. However, these issues have not been systematically investigated and it is not clear what the most effective value, or format, of HPFI is.

## Conclusion

The available evidence from controlled studies suggests that HPFI are more effective than usual care or no intervention at encouraging healthy behaviour change amongst non-clinical adult populations living in high income countries. There was not convincing evidence that HPFI are more effective for ‘simple’ compared to ‘complex’ behaviours. There was some evidence that effects decrease as post-intervention follow-up increases and as incentive value increases. However, the available evidence is substantially limited, particularly in relation to the range of behaviours studied.

## Supporting Information

File S1 Protocol.
(PDF)

File S2 Search strategy for identification of studies for financial incentives.
(PDF)

Table S1 Inclusion criteria.
(PDF)

Table S2 Characteristics of included studies.
(PDF)

Checklist S1 PRISMA checklist.
(PDF)
