# Supplementary material for: The Effectiveness of Financial Incentives for Health Behaviour Change: Systematic Review and Meta-Analysis
Source: PLoS One. 2014 Mar 11;9(3):e90347. doi: 10.1371/journal.pone.0090347 (PMC3949711; doi:10.1371/journal.pone.0090347)
Supplement: File S1 — Protocol. (PDF) [file pone.0090347.s001.pdf]

PROSPERO International prospective register of systematic reviews

A systematic review of the use of financial incentives to encourage uptake of healthy behaviours

Emma L Giles, Jean Adams, Falko F Sniehotta, Elaine McColl, Shannon Robalino

Citation

Emma L Giles, Jean Adams, Falko F Sniehotta, Elaine McColl, Shannon Robalino. A systematic review of the use of financial incentives to encourage uptake of healthy behaviours. PROSPERO 2012:CRD42012002393 Available from http://www.crd.york.ac.uk/PROSPERO_REBRANDING/display_record.asp?ID=CRD42012002393

Review question(s)

Primary research question:

What is the effectiveness of financial incentives for encouraging uptake of healthy behaviours amongst adults living in high-income countries?

Additional research questions:

1. What formats do the financial incentives take (e.g. cash, coupons, vouchers, pay deductions)?

2. What is the range of healthy behaviours that have been targeted (e.g. smoking, physical activity, dietary behaviours, alcohol consumption, safe sun, safe sex, and primary preventive clinical behaviours)?

3. Apart from the incentive component, what other behaviour change techniques are used as part of these interventions?

4. What theoretical rationales have been used to guide intervention development?

5. Does effectiveness of financial incentive programmes vary according to:

a. the length of the intervention period?

b. the length of the follow up period, after the active intervention ceases?

c. the format of the incentive (e.g. cash, voucher, reward, penalty, total value)?

d. the nature of the behaviour incentivised (e.g. one-off, sustained behaviour change)?

e. socio-demographic characteristics (e.g. age, gender, socio-economic position) of recipients?

f. inclusion of other behaviour change techniques in the intervention programme?

Searches

Search strategies:

Both published and unpublished, or grey, literature will be included in the systematic review. The following sources will be searched to identify published literature:

• Electronic databases of peer-reviewed journal articles; including those covering biomedicine (e.g. MEDLINE, EMBASE, Science Citation Index), nursing and allied health professions (e.g. CINAHL), and the social sciences (e.g. Social Science Citation Index, PsycINFO, ASSIA (Applied Social Science Index and Abstracts), IBSS (International Bibliography for the Social Sciences)).

• Online research registers; including trial registers (e.g. Current Controlled Trials, clinicaltrials.gov) and systematic review registers (e.g. Cochrane Library of Systematic Reviews, DARE).

• The reference lists of all studies that meet the inclusion criteria, as well as relevant reviews will be scanned to identify further relevant publications.

• Citation searches of all studies that meet the inclusion criteria will be performed in the Science and Social Science Citation Indices to identify other relevant publications.

Search terms will aim to capture the key concepts in the inclusion criteria. The search strategy will take the general form of: incentives AND terms for specific healthy behaviours and will be developed with the help of an information scientist (SR). A process of ‘pearl growing’ will be used whereby keywords of relevant publications found during initial searching will be used to iteratively develop the search strategy. Once the generic search strategy has been finalised, it will be adapted for use in each electronic database to maximise the potential of individual keyword dictionaries.

Ancillary search procedures:

As the inclusion criteria are restricted to RCTs and ITSA it is not anticipated that there will be a large body of grey literature. In addition to searching trial registers, unpublished, or grey, literature will be accessed by sending emails to relevant on-line discussion lists and organisations covering topics including public health and preventive medicine (e.g. public-health@jiscmail.ac.uk; US Preventive Services Task Force), incentives for health (e.g. health-incentives@jiscmail.ac.uk), and evidence synthesis (e.g. Cochrane Effective Practice and Organisation of Care Group, Cochrane Public Health Group, The Joanna Briggs Institute) requesting that any unpublished reports are forwarded to the study team.

Types of study to be included

Study design: randomised controlled trials, cluster randomised controlled trials or interrupted time series analyses (ITSA)

Condition or domain being studied

Health-promoting behaviours include the ‘big four’ behaviours of not smoking, sensible drinking, a ‘healthy’ diet, and regular physical activity; as well as safe sun and sex behaviours and primary preventive clinical behaviours such as attending for vaccinations and screening. Poor engagement in these behaviours is a key determinant of morbidity and mortality and results in substantial social, healthcare and economic costs. Despite consistent efforts to encourage uptake of healthy behaviours, unhealthy behaviours remain common. Developing effective methods to encourage uptake of healthy behaviours will result in substantial benefits to society as a whole.

In general, behavioural incentives have been defined as motivating rewards provided contingent on behavioural performance. However, this definition could be interpreted as including any reward (e.g. a sticker or praise) and not just financial rewards. Furthermore, this definition specifically excludes the converse of motivating rewards - penalties. Whilst non-financial rewards may also increase health promoting behaviours, it is highly likely that in advanced societies, interventions offering financial rewards are conceptually different from those offering rewards with social, emotional or tokenistic value. As such, this review is restricted to financial incentives and we define that as interventions that offer cash or cash-

like (e.g. vouchers that can be exchanged for goods or services) rewards contingent on performance of the target healthy behaviour, but widen this to include interventions imposing cash or cash-like (e.g. reductions in welfare benefits) penalties contingent on non-performance of the target healthy behaviour.

The reasons why individuals pursue unhealthy behaviours are diverse and include environmental social, psychological and socio-demographic factors. One further reason relates to the relative balance and timing of the costs and rewards of engaging in these behaviours, and personal preferences for these (i.e. 'time preference'). Behavioural economic theory suggests that individuals commonly hold inconsistent preferences for similar outcomes occurring at different points in the future, with outcomes in the near future generally valued more than those in the distant future. Whilst the health gains of health-promoting behaviours are often delayed in time, the financial and opportunity costs can be immediate. As these immediate costs are 'dis-valued' more than the delayed health benefits are valued, individuals make a 'rational' choice to pursue unhealthy behaviours. Providing immediate rewards for performance of health behaviours, or penalties for non-performance is likely to change the temporal reward structure associated with these behaviours, working with, rather than against, individuals' time preferences.

Using financial incentives to encourage uptake of healthy behaviours may appear to be a simple solution to a serious public health problem. However, these interventions are not simple. They differ in terms of the behaviour incentivised; the value, nature (i.e. reward or penalty; cash or voucher) and timing (i.e. immediately following, or remote from, performance of the behaviour) of the incentive; as well as the other behavioural change techniques that may be incorporated into the programmes which they are part of (e.g. keeping a record of the target behaviour or agreeing and behavioural contract). Whilst there is a growing range of evaluations of financial incentives for encouraging uptake of healthy behaviours, conclusions on what makes an effective financial incentive programme in this context remain limited to the suggestion that incentives are more useful for encouraging simple one-off behaviours, such as attendance for vaccinations, than more complex sustained behaviour changes, such as smoking cessation. In addition, variations in the effectiveness of financial incentives across population groups have not been sufficiently explored. The apparent failure of many financial incentive programmes to achieve sustained behavioural change may reflect sub-optimal design of the intervention, rather than a failure of incentives per se.

The UK Secretary of State for Health has signalled his interest in using financial incentives for encouraging uptake of health behaviours, and the National Institute for Health and Clinical Excellence is consulting on the topic. This research will provide policy-relevant information for the design of evidence-based financial incentive interventions which can be subjected to robust evaluation.

A number of systematic and non-systematic reviews have been conducted in this area. However, most focus on individual behaviours (e.g. smoking, or weight loss), rather than exploring the full range of healthy behaviours, or are restricted to developing countries where absolute financial hardship may be much more common. The current review will fill this gap by conducting a systematic review of primary studies randomised and non-randomised controlled studies which explore the use of financial incentives to encourage uptake of the full range of healthy behaviours. Explicit procedures will be used to search the literature and identify reports of research for inclusion. Established tools for assessing the quality of included reports will be used to grade the quality of existing evidence. A narrative and tabular summary of the existing evidence will be produced. The potential for meta-analysis will be explored and this will be conducted if appropriate.

This review has been designed using the approach suggested by the EPPI-Centre at the Institute of Education, London. This is consistent with Cochrane methodology. The review will be registered with PROSPERO, the International Prospective Register of Systematic Reviews (www.crd.york.ac.uk/prospero/), once protocol finalisation has occurred, but before searches begin. By providing a lasting record of the review protocol, and any changes to this, prospective registration reduces

the possibility of selective reporting and any associated bias.

Participants/ population

Inclusion criteria.

Research reports will be included in the review if they meet all of the following criteria:

Population: adults (aged 18 years or over) living in high-income economies (those with a Gross National Income of $12,276 or more per capita in 2010, as identified by the World Bank)

Intervention(s), exposure(s)

Intervention: financial incentives designed to encourage uptake of health behaviours at the individual level; defined as: cash or cash-like rewards (or penalties) provided contingent on change (or non-change) in a specific healthy behaviour (e.g. increase in physical activity).

Healthy behaviours will include: smoking cessation; increase in physical activity; decrease in harmful or hazardous drinking; increase in consumption of ‘healthy’ foods such as fruit and vegetables; decrease in consumption of ‘unhealthy’ foods such as those high in fat, salt or sugar; and attendance for screening or vaccination.

Comparator(s)/ control

Comparator: usual care, no intervention or two-factor designs.

Context

Language: Have an English language title and abstract

Date range: All dates

Outcome(s)

Primary outcomes

Outcome measure: objective or self-report measures of healthy behaviour that have been validated against objective measures.

Change in target healthy behaviour.

Secondary outcomes

We include here all other variables of interest: Format of financial incentive; target behaviour; theoretical rationale for development of financial incentive intervention; other behavioural change techniques used alongside financial incentive; length of intervention period; length of follow up period after active intervention has ended; socio-demographic characteristics of participants.

Data extraction, (selection and coding)

After importing search results into EndNote and removing duplicates, screening will be conducted in two phases. Firstly, titles and abstracts will be screened by one researcher (ELG) to identify publications that definitely do not meet the inclusion criteria. These publications will be excluded. In any cases of doubt, publications will be included.

Secondly, the full papers of publications that were included following the first screening will be screened again by the two researchers (ELG and JA), to identify those that definitely do not meet the inclusion criteria. On this occasion the assessment will be whether publications meet the inclusion criteria. Any disagreements at this stage will be resolved by discussion. Tables of excluded studies will be prepared, detailing when exclusion occurred and reasons for exclusion.

Data extraction (selection and coding):

A coding framework will be developed in RevMan 5. This will be used to record details of, as appropriate: participant characteristics, setting, time period, intervention, comparator, outcome, study design, method of analysis, factors considered in analysis, results, and quality assessment. Data will be extracted by one reviewer (ELG) and checked by a second (JA). Any disagreements will be resolved by discussion. The behaviour change techniques used alongside the financial incentives will be assessed using Michie et al's (2011) revised taxonomy of behavioural change techniques20.

As we will make efforts to access grey literature, it is likely that there will be cases where we retrieve both an internal report and a peer-reviewed paper on the same study. In these cases, peer-reviewed findings will be favoured, although additional details provided in the internal report will be extracted if relevant. Where publications lack details required for quality assessment or full data extraction, authors will be contacted to request further details.

Risk of bias (quality) assessment

The quality of all studies that meet the inclusion criteria will be formally assessed. This information will be used to describe the quality of available research and to conduct sensitivity analysis in any meta-analyses. Quality will be assessed by two researchers (JA and EG) working independently using the Cochrane risk of bias tool and an overall assessment of risk of bias for each study made. Any studies with a 'high risk of bias' will be excluded from any meta-analyses during sensitivity analysis.

Strategy for data synthesis

We will begin by describing the range of interventions (both financial incentive and other behaviour change technique components), the theoretical rationales used to guide incentive development, population characteristics, and the behavioural outcomes that have been studied. The length of the intervention and follow-up periods will be presented overall, as well as the nature of the incentive, the nature of the behaviour incentivised and the size of the reported effects. These will be grouped according to population, intervention type and outcome, with the results summarised in tabular form.

The potential for meta-analyses, using random or fixed effects models as appropriate, will be explored and conducted if applicable. If appropriate, separate meta-analyses will be conducted for different types of interventions and outcomes. Funnel plots will be used to explore publication bias. Sensitivity analysis will explore the effect of excluding studies that appear to be statistical outliers, and those of poorer methodological quality. All analyses will be conducted in RevMan 5.

As previously, the number and type of behavioural change techniques used in each intervention included in effectiveness studies will be documented and compared to effect size to determine if any clear patterns emerge.

Finally, we will prepare a 'summary of findings' table as described in the Cochrane Handbook and use the GRADE approach for describing the quality of relevant evidence.

Analysis of subgroups or subsets

None planned.

Dissemination plans

In order to disseminate our findings to the academic community, we will write up and submit our results for

publication in a peer-review journal (e.g. Health Psychology). We will also present our results at a meeting of a learned society (e.g. UK Society for Behavioural Medicine). To disseminate our findings to members of the public health policy and practice community, we will produce distribute an evidence briefing and present our work at a regional meeting of Fuse – the Centre for Translational Research in Public Health. Such meetings routinely attract audiences of more than 100 members of the public health research, policy and practice community from across the North East.

Contact details for further information

Emma L Giles

Institute of Health and Society

3rd Floor Baddiley-Clark Building

Newcastle University

Newcastle upon Tyne

Tyne and Wear, NE2 4AX

e.l.giles@ncl.ac.uk

Organisational affiliation of the review

Newcastle University

www.ncl.ac.uk

Review team

Dr Emma L Giles, Newcastle University
Dr Jean Adams, Newcastle University
Dr Falko F Sniehotta, Newcastle University
Professor Elaine McColl, Newcastle University
Ms Shannon Robalino, Newcastle University

Anticipated or actual start date

02 January 2012

Anticipated completion date

30 June 2013

Funding sources/sponsors

This work is produced under the terms of a Career Development Fellowship research training fellowship issued by the NIHR to Dr Jean Adams.

Dr Jean Adams and Dr Emma Giles are funded in part, and Dr Falko Sniehotta is funded in full, as staff members of FUSE, the Centre for Translational Research in Public Health, a UKCRC Public Health Research Centre of Excellence. Funding for FUSE from the British Heart Foundation, Cancer Research UK, Economic and Social Research Council, Medical Research Council, the National Institute for Health Research, under the auspices of the UK Clinical Research Collaboration is gratefully acknowledged.

Conflicts of interest

None known

Other registration details

Additional registration is being sought with BMC Public Health (not yet confirmed).

Language
English

Country
England

Subject index terms status
Subject indexing assigned by CRD

Subject index terms
Financing, Personal; Health Behavior; Health Promotion; Humans; Motivation; Patient Compliance

Reference and/or URL for protocol
http://www.crd.york.ac.uk/PROSPEROFILES/2393_PROTOCOL_20120416.pdf

Date of registration in PROSPERO
14 May 2012

Date of publication of this revision
02 October 2012

Stage of review at time of this submission	Started	Completed
Preliminary searches	Yes	No
Piloting of the study selection process	Yes	No
Formal screening of search results against eligibility criteria	Yes	No
Data extraction	Yes	No
Risk of bias (quality) assessment	Yes	No
Data analysis	No	No

PROSPERO
International prospective register of systematic reviews
The information in this record has been provided by the named contact for this review. CRD has accepted this information in good faith and registered the review in PROSPERO. CRD bears no responsibility or liability for the content of this registration record, any associated files or external websites.
