# Supplementary material for: The Effectiveness of Financial Incentives for Health Behaviour Change: Systematic Review and Meta-Analysis
Source: PLoS One. 2014 Mar 11;9(3):e90347. doi: 10.1371/journal.pone.0090347 (PMC3949711; doi:10.1371/journal.pone.0090347)
Supplement: Table S1 — Inclusion criteria. (PDF) [file pone.0090347.s003.pdf]

Table S1 Inclusion criteria

Study component	Inclusion criteria
Study design	Randomized controlled trials, cluster randomized controlled trials, controlled before and after studies or interrupted time series analyses
Publication language	All languages (English language title and abstract)
Population	Non-clinical, adult (at least 50% of the sample aged 18 years or above) populations, living in high-income economies (those with a Gross National Income of $12,276 or more per capita in 2010, as identified by the World Bank)
Intervention	Financial incentives: cash, cash-like rewards, or penalties contingent on behaviour change or non-change
Target behaviours	Smoking cessation, physical activity, alcohol consumption, safe sun behaviours, safe sex behaviours, healthy food behaviours, attendance for screening or vaccination
Comparators	Usual care, no intervention
Outcome measures	Objective or validated self-reported measures
