# Supplementary material for: The Effectiveness of Financial Incentives for Health Behaviour Change: Systematic Review and Meta-Analysis
Source: PLoS One. 2014 Mar 11;9(3):e90347. doi: 10.1371/journal.pone.0090347 (PMC3949711; doi:10.1371/journal.pone.0090347)
Supplement: Table S2 — Characteristics of included studies. (PDF) [file pone.0090347.s004.pdf]

Table S2 Characteristics of included studies

Paper	Participants recruited	Incentive intervention	Additional intervention arms	Comparator	Outcome & measurement	Study design & follow-ups (from baseline)	Results
**Smoking Cessation**							
Donatelle et al 2000[48]	Pregnant women cigarette smokers living in Oregon, USA aged 15 years or older; 112 in intervention group, 108 in control group.	*Direction:* reward<br>*Form:* vouchers for restricted range of goods/services (unknown voucher types)<br>*Magnitude (Total maximum value of incentive payments 2011 $US):* $653<br>*Certainty:* certain<br>*Target:* outcome (voucher for biochemical evidence of smoking cessation)<br>*Frequency:* some instances incentive (when cessation confirmed)<br>*Immediacy:* at baseline, eight months gestation & two months	None	Usual care (smoking cessation self-help kit)	Smoking cessation measured using salivary cotinine <30ng/ml confirmed with salivary thiocyanate <100ug/ml.	Two-arm RCT with follow-ups at 8 months gestation (105 in intervention group, 102 in control group); and 2 months post-partum (103 in incentive group, 102 in control group).	Smoking cessation significantly higher in intervention vs. control group at 8 months gestation and 2 months post-partum.<br>Smoking cessation rate at 8-months gestation 32% in intervention group vs. 9% in control group (p<0.0001). Smoking cessation rate at 2-months post-partum 21% in intervention

		postpartum<br>*Schedule:* fixed<br>*Recipient:* individuals<br>*Recruitment setting:* community<br>*Delivery setting:* community<br>*Length of intervention from baseline*: up to 10 months					group and 6% in control group (<0.001).
Glasgow et al 1993[50]	Adult smokers living in Salem & Portland, Oregon, USA; 474 in intervention group, 623 in control group.	*Direction:* reward<br>*Form:* cash (for certain component) & specific goods/services (for uncertain change component)<br>*Magnitude (Total maximum value of incentive payments 2011 $US):* $187 (certain component), at least $965 (chance component)<br>*Certainty:* certain & uncertain chance<br>*Target:* outcome (cash for biochemical evidence of smoking cessation plus entry into monthly	None	No intervention	Smoking cessation measured using expired CO <9ppm.	Two-arm cluster RCT, with clustering of workplaces, with follow-ups at 12 months (381 in intervention group, 475 in control group) and at 24 months (344 in intervention group, 426 in control	No difference in smoking cessation between intervention and control group at 12 or 24 months.<br>Smoking cessation rate at 12 months 11% in intervention group vs. 12% in control (p>0.05). Smoking cessation rate at 24 months 14% in intervention group and 12% in control group.

		sweepstakes)<br>*Frequency:* some instances incentivised (when cessation confirmed)<br>*Immediacy:* at monthly check-in meetings<br>*Schedule:* fixed<br>*Recipient:* individuals<br>*Recruitment setting:* workplace<br>*Delivery setting:* workplace<br>*Length of intervention period from baseline*: 12 months				group).	
Jason et al 1995; 1997[51, 52]	Adult smokers living in Chicago, USA; 283 in incentive plus group sessions group, 281 in incentive only intervention group, 280	*Direction:* reward<br>*Form:* cash<br>*Magnitude (Total maximum value of incentive payments 2011 $US):* $245<br>*Certainty:* certain<br>*Target:* outcome<br>*Frequency:* some instances incentivised (each day abstinent)	Two intervention arms: incentives alone (group 1); incentive plus group sessions (group 2).	Usual care (self-help manual)	Smoking cessation measured using expired CO <9ppm, confirmed with salivary cotinine.	Three-arm cluster RCT, with clustering of workplaces, with follow-up at 6, 12, 18 and 24 months; those lost to follow up assumed	Smoking cessation significantly higher in group 2 vs. group 1; and group 1 vs. control group.<br>Smoking cessation rate at 6 months 25% in group 2 vs. 10% in group 1 vs. 4% in control

	in (self-help) control group.	*Immediacy:* unclear<br>*Schedule:* fixed<br>*Recipient:* individuals<br>*.Recruitment setting:* workplace<br>*Delivery setting:* workplace<br>*Length of intervention period from baseline:* initial 3 week period, then over 6 months				not to have quit.	group (p<0.001).<br>Smoking cessation rate at 12 months 21% in group 2 vs. 10% in group 1 vs. 7% in control group (p<0.001).<br>Smoking cessation rate at 18 months 16% in group 2 vs. 12% in group 1 vs. 7% in control group (p value reported as not significant).<br>Smoking cessation rate at 24 months 18% in group 2 vs. 13% in group 1 vs. 10% in control group (p value reported as not significant).
Jeffery 1990[53]	Adult smokers living in Minnesota	*Direction:* penalty<br>*Form:* cash<br>*Magnitude (Total maximum value of*	None	Usual care (newsletter on smoking cessation)	Smoking cessation measured using salivary	Two-arm RCT, with follow-up at 6 months (6	Smoking cessation higher in intervention vs. control group.

	USA; 133 in intervention group, 9 in control group.	*incentive deductions 2011 $US):* $103<br>*Certainty:* certain<br>*Target:* outcome<br>*Frequency:* some instances incentivised (each month abstinent)<br>*Immediacy:* at the end of the 6-month program<br>*Schedule:* fixed<br>*Recipient:* individuals<br>*Recruitment setting:* community – not workplace<br>*Delivery setting:* community – not workplace<br>*Length of intervention period from baseline:* 6 months			cotinine <11ng/ml	in intervention group, 118 in control group).	No tests of significance reported.<br>Smoking cessation rate at 6 months 20% in intervention group vs. 9% in control group.
Jeffery et al 1993[54]	Adult smokers living in Minneapolis, USA; 9549 in intervention	*Direction:* penalty<br>*Form:* cash<br>*Magnitude (Total value of incentive deductions 2011 $US):* $342<br>*Certainty:* certain	None	No intervention	Smoking cessation measured using expired CO <8ppm, confirmed.	Two-arm cluster RCT, with clustering of workplaces, with follow-	Smoking cessation higher in control vs. intervention group.<br>Smoking cessation rate at

	group, 10322 in control group.	*Target:* outcome<br>*Frequency:* some instances incentivised (when cessation confirmed)<br>*Immediacy:* immediately<br>*Schedule:* fixed<br>*Recipient:* individual<br>*Recruitment setting:* community – workplace<br>*Delivery setting:* community – workplace<br>*Length of intervention period from baseline:* 24 months				up at 24 months; those lost to follow up assumed not to have quit.	24 months 21.71% in intervention vs. 22.82% in control group.
Stitzer and Bigelow 1983[59]	Adult smokers; 14 in intervention group 1, 16 in $intervention group 2, 14 in intervention group 3, 15 in control	*Direction:* reward<br>*Form:* cash<br>*Magnitude (Total value of incentive payments 2011 $US):* $22.60-$226 (depending on intervention group)<br>*Certainty:* certain<br>*Target:* outcome<br>*Frequency:* some instances incentivised	Three intervention arms: $1/day (group 1), $5/day (group 2), $10/day (group 3).	No intervention	Smoking cessation using a CO cut-off based on 50% of average baseline smoking behaviour.	Four-arm RCT, with follow-up at 4 weeks; those lost to follow up assumed not to have quit.	The adjusted average number of daytime cigarettes decreased as incentives increase, p<0.001.<br>Success at meeting smoking targets: 28.6% in group 1, 56.3%

	group.	(each day abstinent)<br>*Immediacy:* immediately<br>*Schedule:* fixed<br>*Recipient:* individual<br>*Recruitment setting:* community – workplace<br>*Delivery setting:* community –workplace<br>*Length of intervention period from baseline:* 2 weeks					in group 2, 78.6% in group 3 and 6.7% in control group.
Stitzer and Bigelow 1985[60]	Adult smokers; 11 in intervention group 1, 8 in intervention group 2, 9 in control group.	*Direction:* reward<br>*Form:* cash<br>*Magnitude (Total value of incentive payments 2011 $US):* $105<br>*Certainty:* certain<br>*Target:* outcome<br>*Frequency:* some instances incentivised (each day abstinent)<br>*Immediacy:* immediately<br>*Schedule:* fixed<br>*Recipient:* individual	Two intervention arms: $5/day for CO readings <8ppm (group 1), $5/day for CO readings <16ppm (group 2).	No intervention	Smoking cessation using CO cut-off points of <8ppm and <16ppm.	Three-arm RCT, with follow-up at 4 weeks; those lost to follow up assumed not to have quit.	Smoking cessation significantly higher in the group 1 vs. group 2 or control group (p<0.003).<br>Success at meeting smoking cessation targets: 45.5% in group 1, 87.5% in group 2, and 0% in control group.

		Recruitment setting: community – workplace<br>*Delivery setting:* community - workplace<br>*Length of intervention period from baseline:* 2 weeks					
Volpp et al 2006[61]	Adult smokers living in Philadelphia, USA; 92 in intervention group, 87 in control group.	*Direction:* reward<br>*Form:* cash<br>*Magnitude (Total value of incentive payments 2011 $US):* $223<br>*Certainty:* certain<br>*Target:* process (attendance at sessions) & outcome (for cessation)<br>*Frequency:* some instances incentivised (when cessation confirmed)<br>*Immediacy:* immediately<br>*Schedule:* fixed<br>*Recipient:* individual<br>*Recruitment setting:* outpatient	None	Usual care	Smoking cessation verified by urine cotinine test <500 ug/ml.	Two-arm RCT, with follow-up at 14 weeks and 36 weeks; those lost to follow up assumed not to have quit.	Smoking cessation significantly higher at 30-days in intervention vs. control group (p=0.01). No difference at 6 months (p=0.57).<br>Smoking cessation 16.3% in intervention group, 4.6% in control group at 30-days; and 6.5% in intervention group, 4.6% in control group at 6 months.

		Delivery setting: outpatient<br>*Length of intervention period from baseline:* 10 weeks					
Volpp et al 2009[62]	Adult smokers, living in the US; 436 in intervention group, 442 in control group.	*Direction:* reward<br>*Form:* cash<br>*Magnitude (Total value of incentive payments 2011 $US):* $786<br>*Certainty:* certain<br>*Target:* process (sessions, participation & samples) & outcome (cessation)<br>*Frequency:* some instances incentivised (when cessation confirmed)<br>*Immediacy:* immediately<br>*Schedule:* fixed<br>*Recipient:* individual<br>*Recruitment setting:* community – workplace<br>*Delivery setting:* community – workplace	None	Usual care	Smoking cessation verified by saliva cotinine measure of <15ng/ml or urine cotinine <2ng/ml	Two-arm RCT, with follow-up at 3 or 6 months, 9 or 12 months and 15 or 18 months; those lost to follow up assumed not to have quit.	Smoking cessation significantly higher in intervention vs. control group.<br>Cessation at 3 or 6 months was 20.9% in the intervention group, vs. 11.8% in the control group (p<0.001); at 9 or 12 months 14.7% in the intervention group vs. 5% in the control group (p<0.001); at 15 or 18 months 9.4% in the intervention group vs. 3.6% in the control group (p<0.001).

		Length of intervention period from baseline: at least 6 months, maximum of 12 months					
Windsor 1988[63]	Adult smokers living in Birmingham, USA; 95 in intervention group 1, 94 in intervention group 2 and 95 in control group.	*Direction:* reward<br>*Form:* cash<br>*Magnitude (Total value of incentive payments 2011 $US):* $95.10<br>*Certainty:* certain<br>*Target:* outcome<br>*Frequency:* some instances incentivised (when cessation verified)<br>*Immediacy:* at 6 weeks and at 6 months<br>*Schedule:* fixed<br>*Recipient:* individual<br>*Recruitment setting:* community – workplace<br>*Delivery setting:* community – workplace<br>*Length of intervention period from baseline:* 6 months	Four intervention arms: self-help & skills training (group 1); self-help & incentives (group 2); self-help, skills training & incentives (group 3)	Usual care (self-help manual only)	Smoking cessation verified by salivary thiocyanate <100µg/ml.	Four-arm RCT, with follow-up at 6 weeks, 6 months and 12 months; those lost to follow up assumed not to have quit.	Inconsistent results shown at the 6-week follow-up, and so it was concluded that the monetary incentives would not have an effect at 6 months.<br>Chi-square analysis revealed no significant difference between control and group 2 (p=0.347) or between groups 1 and 3 (p=0.306) at the 6-week point. Smoking cessation at 6 months control

							7%, Group 1 8%, Group 2 20%, Group 3 12%; at 12 months control 6%, Group 1 5%, Group 2 19% and Group 3 5%.
**Vaccination & Screening**							
Debari and Servodidio 2007[47]	Adults attending for breast and cervical cancer screening, living in Connecticut, USA; unknown participant numbers.	*Direction:* reward<br>*Form:* vouchers for restricted range of goods/services<br>*Magnitude (Total value of incentive payments 2011 $US):* $5.42<br>*Certainty:* certain<br>*Target*: process (maintained appointment or not)<br>*Frequency:* all instances incentivised (one-off)<br>*Immediacy:* on attendance<br>*Schedule:* fixed (one-off)<br>*Recipient:* individual<br>*Recruitment setting:*	None	No intervention	Attendance for breast or cervical screening.	Two-arm RCT, with follow-up as one-off attendance.	No difference in attendance rates between intervention vs. control group.<br>Attendance rates or tests of significance not reported.

		outpatient<br>*Delivery setting:* outpatient<br>*Length of intervention period from baseline:* One-off attendance					
Malotte et al 1998[55]	Adults attending for TB skin test reading, living in California, USA; 203 in intervention group 1, 198 in intervention group 2, 100 in control group, 204 in intervention group 4, and 200 in intervention group 5.	*Direction:* reward<br>*Form:* cash<br>*Magnitude (Total value of incentive payments 2011 $US):* $6.90-$13.80 (depending on intervention group)<br>*Certainty:* certain<br>*Target:* process (education session) & outcome (attendance)<br>*Frequency:* all instances incentivised (one-off)<br>*Immediacy:* on attendance<br>*Schedule:* fixed (one-off)<br>*Recipient:* individual<br>*Recruitment setting:* community – not workplace	Five intervention arms: $10 plus motivational education (group 1), $5 plus motivational education (group 2), $5 only (group 4), $10 only (group 5).	No intervention.	Attendance for TB skin test reading	Six-arm RCT, with follow-up as one-off attendance.	Attendance was higher for those receiving an incentive (significance tests not reported).<br>Attendance: 92.1% in group 1, 84.3% in group 2, 34.3% in group 3, 33% in control group, 85.8% in group 4, 93% in group 5.

		Delivery setting: community – not workplace<br>*Length of intervention period from baseline:* return for skin test reading within four days (96 hours)					
Malotte et al 1999[56]	Adults attending for TB skin test reading, living in California, USA; 217 in intervention group 1, 217 in intervention group 2, 218 in intervention group 3, and 215 in control group.	*Direction:* reward<br>*Form:* cash or vouchers for restricted range of goods/services<br>*Magnitude (Total value of incentive payments 2011 $US):* $$13.50<br>*Certainty:* certain<br>*Target:* process (education session) & outcome (attendance)<br>*Frequency:* all instances incentivised (one-off)<br>*Immediacy:* on attendance<br>*Schedule:* fixed (one-off)<br>*Recipient:* individual	Four intervention arms: $10 cash (group 1), $10 grocery store coupon (group 2), $10 bus pass or fast-food coupon (group 3).	No intervention	Attendance for TB skin test reading	Five-arm RCT, with follow-up as one-off attendance.	Statistically significant differences in attendance were found between group 1 and group 2 (p=0.002) and between group 1 and group 3 (p<0.001).<br>Attendance: group 1 94.9%, group 2 85.7%, group 3 82.6%, control 49.3%.

		Recruitment setting: community – not workplace<br>*Delivery setting:* community – not workplace<br>*Length of intervention period from baseline:* return for skin test reading within four days (96 hours)					
Nowalk et al 2010[57]	Adults attending for influenza vaccination. Appears to be a US study; 3757 in control group, 4387 in intervention group.	*Direction:* reward<br>*Form:* vouchers for restricted range of goods/services<br>*Magnitude (Total value of incentive payments 2011 $US):* $5.16<br>*Certainty:* certain<br>*Target:* outcome<br>*Frequency:* all instances incentivised (one-off)<br>*Immediacy:* on attendance<br>*Schedule:* fixed (one-off)<br>*Recipient:* individual	None	Usual care (influenza advertising)	Attendance for influenza vaccination (change in vaccination rate from baseline to intervention year).	Three arm cluster RCT; with follow-up as one-off attendance.	Average change in vaccination rate from previous to current year significant in intervention group (p<0.005).<br>Attendance for vaccination: 11.1% in intervention group, 4.7% in control group.

		Recruitment setting: community – workplace<br>*Delivery setting:* community – workplace<br>*Length of intervention period from baseline:* return for influenza vaccination within 12 month period					
Stitzer 2010[58]	Adults attending for Hepatitis B vaccination, living in the USA; 13 in intervention group, 13 in control group.	*Direction:* reward<br>*Form:* cash & specific goods/services<br>*Magnitude (Total value of incentive payments 2011 $US):* $273 (certain component), at least $16,800 (chance component)<br>*Certainty:* certain & certain chance<br>*Target:* process (study procedures) & outcome (vaccinations)<br>*Frequency:* all instances incentivised (on attendance)<br>*Immediacy:* on	None	Usual care	Attendance for vaccination series.	Two-arm RCT; with follow-up as attendance at vaccination series.	Attendance for all injections was significantly higher in intervention vs. control group (p=0.107).<br>Attendance for all injections in incentive group 77% vs. control 46%.

		attendance<br>*Schedule:* variable<br>*Recipient:* individual<br>*Recruitment setting:* community – not workplace & clinical - outpatient<br>*Delivery setting:* community – not workplace<br>*Length of intervention period from baseline:* over 24 weeks (injections at weeks 2, 4, 8, 12, 16, 20)					
**Physical Activity**							
Finkelstein et al 2008[49]	Adults participating in a physical activity intervention, living in North Carolina, USA; 35 in intervention group, 35 in control group.	*Direction:* reward<br>*Form:* cash<br>*Magnitude (Total value of incentive payments 2011 $US):* $104<br>*Certainty:* certain<br>*Target:* process (attendance at meeting) & outcome (behaviour)<br>*Frequency:* all instances incentivised	None	No intervention	Wearing a pedometer and logging minutes of physical activity.	Two-arm RCT; with follow-up at the end of the four-week intervention period (21 in incentive group, 30 in control group).	Controlling for socio-demographics, significantly more daily physical activity in intervention vs. control group (p<0.001).<br>Minutes of physical activity per week: 35 minutes in

		(using a pedometer)<br>*Immediacy:* each week<br>*Schedule:* variable<br>*Recipient:* individual<br>*Recruitment setting:* community – not workplace<br>*Delivery setting:* community – not workplace<br>*Length of intervention period from baseline:* four weeks					intervention groups vs. 19.5 minutes in control group.
